# Low-cost suture simulator to gain basic surgical skills

**DOI:** 10.1590/acb384223

**Published:** 2023-10-13

**Authors:** Nyara Rodrigues Conde de Almeida, Joyce Pantoja Braga, Lívia Guerreiro de Barros Bentes, Rafael Silva Lemos, Manuela Rodrigues Neiva Fernandes, Gabrielly Leite Andrade, Victor Matheus Mendonça de Araújo, Deivid Ramos dos Santos, Edson Yuzur Yasojima

**Affiliations:** 1 Universidade Federal do Pará – Faculdade de Medicina – Belém (Pará) – Brazil.; 2 Universidade do Estado do Pará – Laboratório de Cirurgia Experimental – Belém (Pará) – Brazil.

**Keywords:** Simulation Training, Low Cost Technology, Experimental Development

## Abstract

**Purpose:**

To introduce a new low-cost simulation model for training basic surgical skills.

**Methods:**

The training model was made from a mixture of 20 g of acetic silicone with 11 g of maize starch. Validation consisted of serial training sessions, evaluating the mean pre- and post-training time and the mean final score according to the global rating scale.

**Results:**

A decrease in the time required to perform the sutures was observed, comparing the average post and pre-training time of each training day, with a significant correlation between the order of training and the time for performing the simulation.

**Conclusions:**

The presented model proved to be capable of simulating the basic suture training skills. It is easy to make, has low cost, and can be easily reproduced in educational institutions.

## Introduction

In its theoretical and practical context, the teaching-learning process of surgical skills constitutes the basis for technical improvement and consequent professional training in medicine^1^,^2^. For many years, the medical training model “see one, do one, teach one” was the basis for teaching surgeons^3^,^4^. With the evolution of evidence-based medicine, this model listed serious points about patient safety, such as non-compliance with surgical techniques, longer hospital stays, and increased morbidity and mortality^4^-^7^.

According to the World Health Organization, patient safety is defined as the reduction, to an acceptable minimum, of the risk of unnecessary damage related to health care. In addition to physical consequences, damage to the patient can lead to increased economic and social costs, reduced achievement of expected results, and greater recurrence of ethical issues^8^. In this sense, knowing that the teaching process of future surgeons is based on the “learning by doing” method, there are several possible situations in which training directly on the patient could result in adverse health events^4^,^9^. Therefore, alternatives for simulated training are sought, replacing models of patients and live animals or human cadavers^1^,^2^,^9^. New student-centered approaches are being established, such as bench-top simulation models, aimed at advancing medical education and increasing performance^1^,^2^,^6^,^7^.

Analyzing training in the surgical area, suturing is one of the basic surgical skills that requires adequate operative technique and movement dexterity. In this high-performance area, the importance of practice-based teaching is understood, with repeated exercises centered on the needs of each student crucial for physical and mental preparation^3^,^6^,^7^. This set guarantees the correct technical execution, coaptation of the wound edges, and operative healing, ruling out possible complications such as dehiscence and infections. However, many of the simulation and training models developed are expensive, with materials that are difficult to access and, therefore, to replicate, which distances students and professionals from their use^1^-^3^,^6^,^9^,^10^.

Thus, it is understood the need to create an experimental model that simulates suturing, a basic surgical skill, that meets the requirements of replacing the practice in humans and reducing infrastructure costs. The study aimed to present a new low-cost and validation simulation and training model for training basic surgical skills.

## Methods

This is an experimental and cross-sectional study, carried out at the Laboratory of Experimental Surgery at the Universidade Estadual do Pará. The research was evaluated and approved by the Research Ethics Committee of the Universidade Estadual do Pará, No. 5.820.060.

### Model making

The training model was made from acetic silicone and corn starch, purchased from a local trade in Belém (PA), Brazil. Initially, with the aid of a digital scale, the proportions of the ingredients were measured and followed by mixing 20 g of acetic silicone with 11 g of maize starch, on a flat non-stick surface, until a homogeneous mass was obtained. Then, the dough was manually molded into a circular shape, with a 6-cm diameter. After its preparation, the model remained in contact with ambient air for 48 hours to dry.

### Model evaluation

At first, the simulator was tested by a surgeon with more than eight years of experience. The test consisted of making a 4-cm longitudinal incision, using a scalpel handle with a 15-blade attached for cutting, followed by making five simple stitches for each type of the following threads: mononylon 6-0 cylindrical needle (16 mm and 3/8 circle), mononylon 5-0 cylindrical needle (16 mm and 3/8 circle), and mononylon 4-0 cylindrical needle (16 mm and 3/8 circle).

After the test, the surgeon was asked general questions about the prototype and its applicability in academia:

- “Can the model be used to train basic suturing skills?”;
- “Is the model easy to use for medical students?”;
- “Does the model allow for gains in technical skill?”;
- “Can the model simulate the characteristics of the skin tissue?”;
- “Is the model easy to replicate and reproduce, considering production time and cost?”.

In addition, questions about the use of different nylon suture, mononylon 4-0, 5-0 and 6-0, were asked:

- “Which of the different options allowed for the best suture and edge coaptation in the proposed model?”;
- “Which of the different suture, after performing manual traction simulating the opening of the wound, showed better resistance to force with a minimum of cracks on the edges of the wound?”.

The following parameters about the model were analyzed:

- Cost of the model;
- Time to make the model;
- Degree of resistance of knots to tensile strength, according to the different types of nylon diameters.

### Model validation

As inclusion criteria for validation of the prototype, medical students attending the 5th academic semester at the Universidade Estadual do Pará, who had not yet attended the discipline of surgical skills and who had not had previous contact with operative synthesis were included. Those who did not agree to sign the Free and Informed Consent Form or who would not be available on the days of the training and evaluation sessions were excluded from the survey. In the end, 20 students met the proposed criteria.

On the first day of planning (D0), all students participated in an expository class given by a general surgeon, totaling 40 minutes in duration, about the concepts and basic skills of surgical suture: presentation of instruments, grip and handling, suture technique, and types of suture. Validation consisted of serial training sessions twice a week for five weeks (D1, D5, D8, D12, D15, D19, D22, D26, D29, D33).

Each simulated training session corresponded to an initial evaluation of the students’ performance time, followed by 1 hour of free training and, in the end, a final evaluation of the performance time. The pre-training and post-training evaluations consisted of carrying out the same operative technique: a 4-cm longitudinal incision, using a scalpel handle with a 15-blade attached for cutting, followed by the suture of five simple knots with mononylon 4-0, cylindrical needle (16 mm and 3/8 circle). During the post-training period of each training day, the students were evaluated by general surgery residents, based on an adaptation of the global rating scale (GRS)^1^,^11^,^12^, an objective scale for assessing individual performance, generating a score from 0 to 35 points (Table 1).

**Table 1:** Global rating scale for student assessment.

Questions	Point scale				
	1	2	3	4	5
1. Respect for tissue	Frequently used unnecessary force on tissue or caused damage by inappropriate use of instruments		Careful handling of tissue but occasionally caused inadvertent damage		Consistently handled tissues appropriately with minimal damage
2. Instrument handling	Repeatedly makes tentative or awkward moves with instruments		Competent use of instruments although occasionally appeared stiff or awkward		Fluid moves with instruments and no awkwardness
3. Motion	Many unnecessary moves		Efficient time/motion but some unnecessary moves		Economy of movement and maximum efficiency
4. Ergonomics	Improper positioningmakes it difficult to perform the procedure		Improper positioning can make it difficult to perform the procedure		Positions perfectly in the operative field
5. Tremors	Presence ofmacroscopic tremors		Tremors that do not affect the performance of the procedure		Absence of fine tremors
6. Suture technique	Unsure, poor technique, and unable to maintain tension		Careful suturing technique and proper tension		Excellent suture controland technique and thecorrect tension
7. Flow of operation	Frequently stoppedoperating or needed to discuss next move		Demonstrated ability for forward planning with steady progression of operative procedure		Planned course of operation with effortless flow from one move to the next
Final score					

Source: Adapted from Martin et al.^11^.

**Figure 1:**
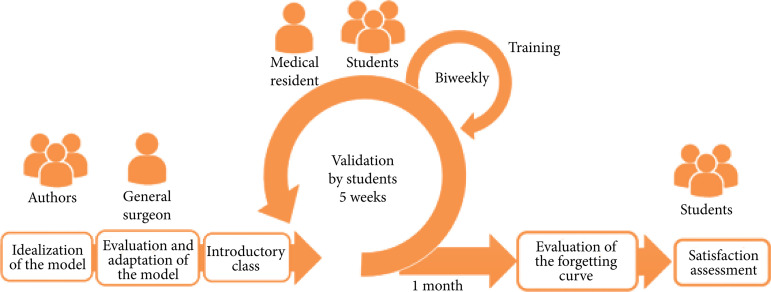
Outline of the research methodology.

Source: Elaborated by the authors.

After a period of four weeks from the last day of training (D61), a new evaluation session of the students was carried out, measuring the performance time for the same described procedure and the grade according to the GRS, to evaluate the performance curve forgetfulness of students (Fig. 1).

The data were presented according to the average time for making the sutures in the pre-training and post-training and according to the average post-training score, on each training day ± standard deviation of the results. Microsoft Word and Excel software were used for data analysis and making graphs and tables and editing photos. Standard descriptive analysis of numerical results was performed, with calculations and measures of central tendency. The Shapiro-Wilk’s normality test was used for quantitative variables. For the comparison of variables, analysis of variance with two factors and repeated measures with Tukey’s post hoc test were used. Comparisons with p ≤ 0.05 were considered significant. Data were tabulated and analyzed using the Jamovi program (Version 2.2.5).

### Participants’ evaluation

In the end, on D61, a simulator evaluation questionnaire was applied, developed, and applied by the Google Forms application, and sent by email of each of the students participants. The questionnaire had a total of 13 statements about the simulator, on a four-point Likert scale:

- Inadequate: 1 point;
- Partially adequate: 2 points;
- Adequate: 3 points;
- Totally adequate: 4 points.

The form’s statements were divided into three general domains, adapted from the study by Pinto et al.^13^:

- Goals: containing four statements about the intended purpose of the model;
- Structure: with five statements referring to form, material, aesthetics, and handling;
- Satisfaction: with four statements related to students’ satisfaction with the training provided by the model.

All participating students completed the questionnaire fully. The data obtained were tabulated and submitted to statistical analysis, calculating the total scores and percentages of approval for each of the 13 statements. The approval averages were calculated for each domain, dividing the sum of their percentages by their respective number of statements. In addition, Cronbach’s alpha index was used to study the form’s reliability and internal consistency, considering a coefficient > 0.75 as validated.

## Results

**Figure 2:**
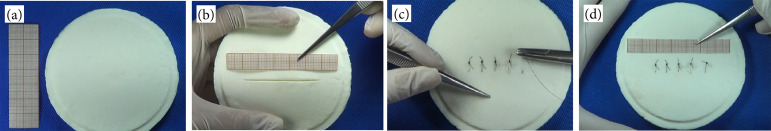
Performing suture training. **(a)** low-cost model; **(b)** longitudinal incision measuring 5 cm; **(c)** the creation of a single stitch suture; **(d)** suture made.

Source: Elaborated by the authors.

The model described proved to be viable for training sutures, ensuring gains and improvements in basic surgical techniques (Fig. 2).

The total cost of the material was R$ 20.4. The average cost of each simulator, proportional to its weight, was less than R$ 1.53 (Table 2). The average time to make the models was 9 minutes, varying between 7 and 13 minutes.

**Table 2:** Costs related to making the model.

Material	Price (R$)	Weight (packaging) (g)	Weight (used) (g)	Price/weight used (R$)
Acetic silicone	16.5	250	20	1.32
Maize starch	3.9	200	11	0.21
Total cost	20.4	Not applicable	1.53	

Source: Elaborated by the authors.

Regarding the surgeon’s assessment, the simulator was considered suitable for making sutures, allowing training in the skill and surgical technique by medical students, in addition to being characterized as easily reproduced, given the low production cost and ease of access to materials. It is a model that does not present difference in texture and thickness that simulates the different layers of human skin tissue, therefore being considered of low fidelity.

During the evaluation of the prototype, manual traction maneuvers were performed on the model, in the longitudinal and transverse directions, to assess the degree of resistance. Analyzing the suture executed with mononylon 6-0, 5-0, and 4-0, the model resisted light and moderate force, with no avulsion or signs of edge tearing. However, when performing higher intensity traction, the tearing up the edges in which were used mononylon 6-0 and 5-0 was observed, an effect not observed with the suture made with mononylon 4-0. It was noted, therefore, that making the suture with a larger diameter wire allows for a greater gain in tensile strength.

**Figure 3:**
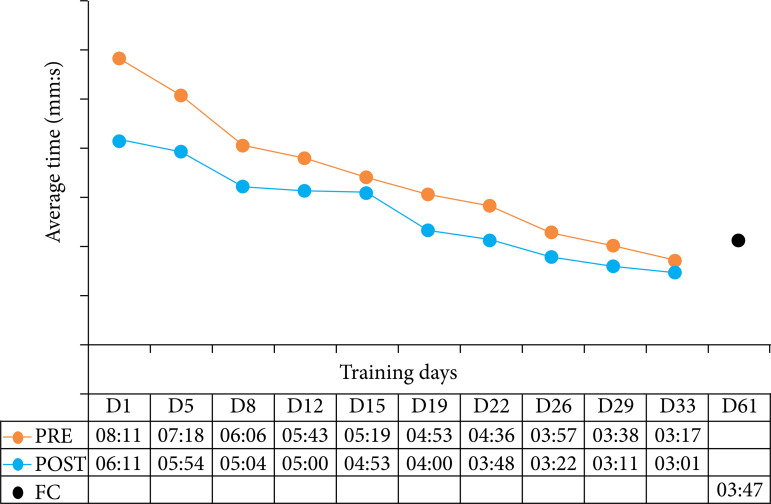
Meantime, in minutes and seconds, in pre- and post-training, and the forgetting curve, according to training days.

PRE: pre-training time; POST: time post-training; FC: forgetting curve. Source: Elaborated by the authors.

Regarding the validation, on the time to perform the five simple stitches, in minutes and seconds, in the pre- and post-training, a decrease in the average time was observed throughout the training, indicating a decreasing line in the graph (Fig. 3). Analyzing statistically, it was possible to perceive a decrease in the time required to perform the sutures, comparing the average time post- and pre-training of each training day, being significant especially the values of the first days of training (D1, D5, D8, D12), returning to significant values in D19, D22, D26 (Table 3).

**Table 3:** Mean time, in minutes and seconds, and ± standard deviation in pre- and post-training, according to training days.

Training day	X ± ST		p-value
	PRE	POST	
D1	08:11 ± 00:42	06:11 ± 00:46	< 0.001
D5	07:18 ± 00:37	05:54 ± 00:19	< 0.001
D8	06:06 ± 00:55	05:04 ± 00:28	< 0.001
D12	05:43 ± 00:29	05:00 ± 00:14	< 0.001
D15	05:19 ± 00:33	04:53 ± 00:28	0.104
D19	04:53 ± 00:56	04:00 ± 00:49	< 0.001
D22	04:36 ± 00:54	03:48 ± 00:18	< 0.001
D26	03:57 ± 00:27	03:22 ± 00:14	0.005
D29	03:38 ± 00:41	03:11 ± 00:37	0.074
D33	03:17 ± 00:35	03:01 ± 00:21	0.953

PRE: pre-training time; POST: post-training time; p < 0.05; ST: standard deviation. Source: Elaborated by the authors.

In addition, it was possible to observe a significant correlation between the training order and the simulation performance time. In this sense, there was a significant decrease in the average time required to perform the suture when comparing the initial pre- and post-training times on D1 with the average pre- and post-training times on the last day of serial training, D33 (p < 0.05). Likewise, when comparing the average times of D1 with the average single time of D61, it was still possible to perceive a significant reduction in the time required to carry out the simulation, even after the interval period without training, marking the forgetting curve (Table 4).

**Table 4:** Mean time, in minutes and seconds, and ± standard deviation in pre- and post-training, comparing the first day of training (D1) with the final training after five weeks (D33) and with the single average time (D61).

	Training day	X ± ST	Training day	X ± ST	p-value
PRE	D1	08:11 ± 00:42	D33	03:17 ± 00:35	< 0.001
POST	D1	06:11 ± 00:46	D33	03:01 ± 00:21	< 0.001
PRE	D1	08:11 ± 00:42	D61	03:47 ± 00:55	< 0.001
POST	D1	06:11 ± 00:46			< 0.001

PRE: pre-training time; POST: post-training time; ST: standard deviation; p-value < 0.05. Source: Elaborated by the authors.

**Figure 4:**
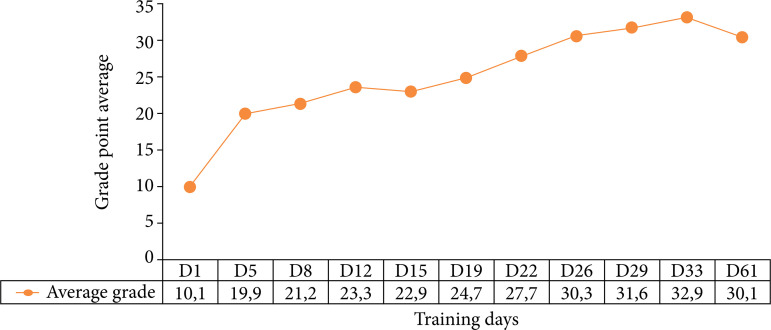
Average score on the global rating scale, according to training days.

Source: Elaborated by the authors.

As for the evaluation of the academics according to the proposed scale, on the first day of the validation test, the final average score among the students was 10.1 ± 0.7. Comparatively, on the last day of training, the students’ final average was 32.9 ± 0.8 (Fig. 4). These values demonstrate the increase in the performance of the students, over the course of the training days, given the increase in the average final score of the participants.

As for the evaluation of the simulator by the students, the “goals” domain obtained the highest approval average among the participants, 99%, the “structure” domain presented an approval average of 97.2%, and the “satisfaction” domain reached 98.7% (Table 5). The internal consistency of the questionnaire, according to the reliability study using Cronbach’s alpha test, reached high values in the three domains, 0.78, 0.941, and 0.889 (Table 6).

**Table 5:** Evaluation of the simulator, based on the goals, structure, and satisfaction domains.

GOALS	Pointing	%Approval
Can be used in academia	100	100
Important for the professional training of academics	100	100
Meets goals of basic suturing skills	98	98
Stimulates interest in surgical skills	98	98
**STRUCTURE**	**Pointing**	**%Approval**
The simulator is easy to understand and practical	100	100
Appropriate for handling medical students	96	96
Simulated training in accordance with the operative technique	96	96
Allows a logical training sequence	100	100
Structures resemble human skin tissue	94	94
**SATISFACTION**	**Pointing**	**%Approval**
Allows training consistent with the level of knowledge of the target audience	98	98
Proposes the construction of knowledge for the target audience	100	100
Allows you to gain lasting technical skills	99	99
Allows reproduction and generalization of basic surgical skill learning	98	98

Source: Adapted from Pinto et al.^19^.

**Table 6:** Simulator evaluation.

Validation criteria	Total score	%Approval	Cronbach
Goals	396	99	0.78
Structure	486	97.2	0.941
Satisfaction	395	98.7	0.889
TOTAL	1.277	98.3	0.899

Source: Elaborated by the authors.

## Discussion

The importance of practice-based teaching has been recognized in several areas of high performance, with repeated exercise being crucial for physical and mental preparation, given the complexity and unpredictable environment of real situations. In surgical teaching, the regimen of practical activities designed and repeated to optimize the improvement and the time of performance can be the fundamental point of mastery of the techniques in the long term^12^-^14^.

In this sense, simulation in the teaching of surgical techniques offers an opportunity to test and improve a wide range of skills, in a controlled and risk-free environment. This scenario allows individualization of teaching, in which the learner develops his skills at his own pace and according to his demand, as well as allowing a mean of evaluating and verifying the simulation performed^15^-^19^. Thus, the present model guarantees simulated training to gain and improve initial surgical skills, valuing the result that, the more repetitions of training, the less time is needed to make the suture.

A simulated training of surgical techniques concerns the need for great logistics, that is, organization of teaching centers, adhesion, training of the teaching staff, and, mainly, technological and high budget demand^15^. Although these points are substantial, the time savings in surgical procedures, especially the reduction of the risks of technical errors and, consequently, the potential for improving patient safety may justify the need to complement simulated teaching in the traditional teaching methodology of future surgeons, see one, do one, teach one^4^,^14^-^16^,^18^,^20^,^21^.

In an attempt to circumvent these limitations, the described model translates into an easy-to-reproduce, low-cost, portable simulator that can be reused, in addition to dispensing with the experimental use of animals, which allows the reproducibility of the surgical suture technique, seeking the introductory practical exercise and skill improvement by repeated training. Likewise, other low-fidelity bench models described in the literature and widely used, such as foam and ethylene vinyl acetate, also proved to be suitable for training surgical skills, reaffirming that technical skills are developed through repetition^1^,^19^,^21^,^22^.

It should also be noted that evaluation and validation are fundamental steps in the process of simulator reliability^17^-^19^. The training format with two weekly sessions is similar to considerable courses internationally, and several works demonstrate that the gain of skills is greater in training with intervals between sessions compared to consecutive activities^23^-^25^. When it comes to validating the presented prototype, when comparing the average overall performance score over the serial training sessions, it was possible to see an increase in the score, reflecting the increased performance of the participants, according to the criteria evaluated in the global classification scale, demarcating, therefore, that repeated training is also related to the evolution of skills and competencies necessary for correct practical execution – correct positioning and ergonomics, familiarity with materials, safety, and motor dexterity^23^-^25^.

Another important point in the validation of the prototype refers to the time it takes to perform the sutures. There was an important reduction in the time required for manufacturing when analyzing the general average time per day of training and the comparison between the average time of the first and last day of the evaluation. When evaluating only the training day after four weeks of interval (D64), it was possible to observe the increase in time, but still significantly lower than the D1 of the experiment. It is understood that basic surgical skills and abilities are developed quickly, considering the first moment of contact with training, followed by a period of stabilization and performance decline^24^,^25^. This scenario proves that basic skills training needs serial repetitions to improve and maintain expertise, demarcating the maintenance of the learning curve^24^-^27^. Thus, the present validation marked the proposed simulation model as suitable for reproducibility, allowing repeated training with continued reassessments, without limitations of place, time, and material.

Finally, the evaluation of the simulation by the participants was carried out using domains recognized in national references of education and validation and authentication with statistical analysis^19^,^28^. In general, the domains had the objective of verifying the desired goals with the simulator, its format related to the technical aspects, and the educational significance of the technology^18^,^19^,^27^,^28^. Thus, all Cronbach’s alpha test values were significant, demonstrating the reliability of the questionnaire as a means of evaluation and, consequently, the validation of the artificial skin simulator as a technical learning tool and the repercussion of knowledge for the scientific community.

The main limitation of the presented model is the median fidelity of the layers and texture of the skin, which decreases the perception of vision about the depth of transfixion of the wound edges. However, this limitation does not make the simulator unusable, as it can be widely used in the initial stages of surgical training, proven to be useful in gaining the skills of the evaluated students, as well as other low-fidelity models already established in the literature^21^,^22^. Finally, it should be noted that, despite the evaluation and validation of the prototype having been carried out with a single stitch suture, the simulator allows the performance of other suture stitches, including continuous stitches.

## Conclusion

The present training model proved to be capable of simulating the basic suture training skills, with easy acquisition and manufacture, as well as low cost. It was also understood that its portability overcomes the restriction of training being done only in specialized centers, demonstrating that the prototype can be easily reproduced in educational institutions. Finally, the proposed model allowed the gain and improvement of skills and competencies for suturing.

## Data Availability

All data sets were generated or analyzed in the current study.
